# The landscape model: A model for exploring trade-offs between agricultural production and the environment

**DOI:** 10.1016/j.scitotenv.2017.07.193

**Published:** 2017-12-31

**Authors:** Kevin Coleman, Shibu E. Muhammed, Alice E. Milne, Lindsay C. Todman, A. Gordon Dailey, Margaret J. Glendining, Andrew P. Whitmore

**Affiliations:** a Sustainable Agriculture Sciences Department, Rothamsted Research, Harpenden, Hertfordshire AL5 2JQ, UK; b Computational and Analytical Sciences Department, Rothamsted Research, Harpenden, Hertfordshire AL5 2JQ, UK

**Keywords:** Modelling, Crops, Soil processes, Nutrient flow, Water movement, Agriculture

## Abstract

We describe a model framework that simulates spatial and temporal interactions in agricultural landscapes and that can be used to explore trade-offs between production and environment so helping to determine solutions to the problems of sustainable food production. Here we focus on models of agricultural production, water movement and nutrient flow in a landscape. We validate these models against data from two long-term experiments, (the first a continuous wheat experiment and the other a permanent grass-land experiment) and an experiment where water and nutrient flow are measured from isolated catchments. The model simulated wheat yield (RMSE 20.3–28.6%), grain N (RMSE 21.3–42.5%) and P (RMSE 20.2–29% excluding the nil N plots), and total soil organic carbon particularly well (RMSE 3.1 − 13.8 %), the simulations of water flow were also reasonable (RMSE 180.36 and 226.02%). We illustrate the use of our model framework to explore trade-offs between production and nutrient losses.

## Highlights

• Understanding trade-offs between yield and environment is essential for SI.
• The Landscape Model aids the understanding of crop-soil-water interactions.
• Model validated against 50 years of data from two long-term experiments.
• Model validated against spatially-explicit data from the North Wyke farm platform.
• The model simulated wheat yield, grain N and grain P particularly well.

## Graphical abstract

**Figure:**
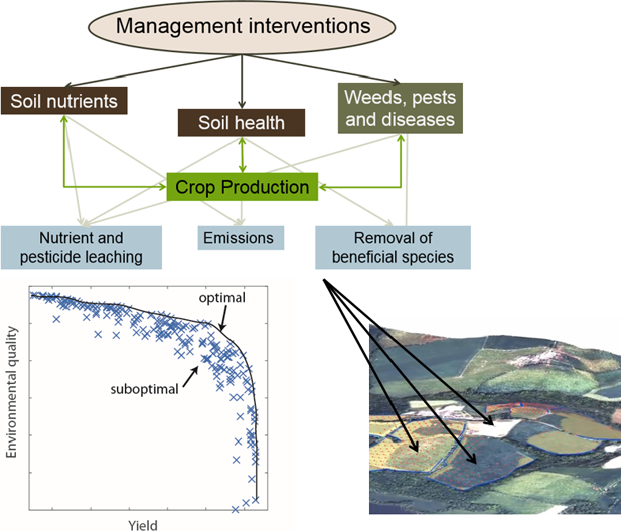

## 1 Introduction

Increasingly, agricultural production is being compelled to look not just at its externalities such as the environmental pollution or depletion of natural resources but also at the provision of wider ecosystem services such as biodiversity. Schemes to monitor or assess land for all of these factors are prohibitively expensive and yet there is a need to analyse modern agricultural systems for the purposes of policy, planning or management. Not surprisingly therefore, computer simulation models have a role to play in filling the large gaps between what we need to know and what is available from measurements.

Simulation models of agricultural systems abound, some focussing on specific aspects such as soil organic matter dynamics (Coleman et al., 1997), crop growth (Semenov and Stratonovitch, 2015), water movement (Addiscott and Whitmore, 1991), emissions (Rolston et al., 1984), competing organisms (Andrew and Storkey, 2017), and some integrating to agricultural management systems (Brisson et al., 2003, Keating et al., 2003). Others focus on the natural systems, tracing biodiversity often quite specifically (Andam et al., 2008, Koh et al., 2010). Some models, particularly agricultural ones, focus on field (Bell et al., 2012, Parton et al., 1994) or farm scales (Del Prado et al., 2011). Biodiversity models often focus on larger scales and water management models are naturally focussed on river basins or catchments (Whitehead et al., 2014).

**Fig 1:**
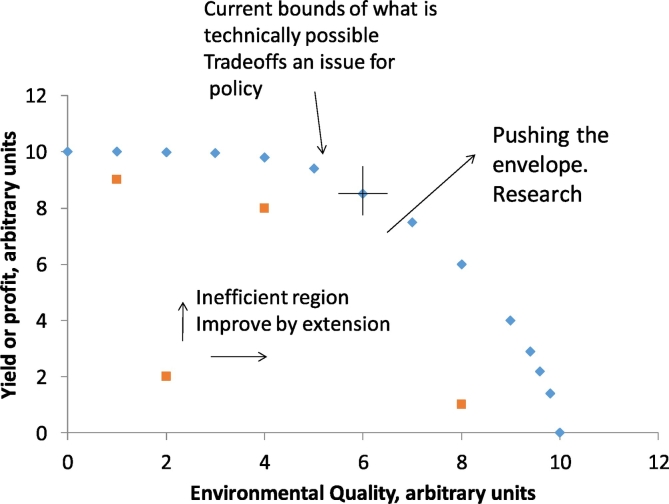
Representation of an environmental-economic production possibility frontier. The blue diamonds are independent outcomes of management that optimises both yield and environmental quality at the same time. A decision along this line is a matter for policy. The orange squares within the envelope are inefficient in the sense that either production or environmental quality could be improved without impacting the other. This is the region for extension. Beyond the envelope is a zone where outcomes are currently infeasible and this is the area which research addresses. An origin placed over any point (for example the cross shown in the figure on the middle of the envelope), facilitates the definition of the envelope algorithmically: if another point can be found in the first quadrant (North East) then the first point in not on the envelope.

Many models simulate fields or regions, some simulate particular fluxes, say water from land to rivers. It is rarer to find models that try to integrate several of the impacts of farming in the landscape, and those that do adopt a relatively empirical, data-driven approach (Jackson et al., 2013, Tilman et al., 2001) that makes it difficult to explore the interactions between components of that landscape that might be better managed with a more holistic overview. It is rarer still to find models that make explicit spatial and temporal linkage between adjacent fields and integrate all aspects of the managed farm environment up to the catchment level. Such a model would be useful to understand the spatial interactions and impact of the natural (weeds, pest and diseases) as well as management (irrigation, fertilizer and application of pesticides) events on an agricultural landscape. Our aim is to develop a spatially explicit model that can simulate the essential processes of soil, water, crop growth and biodiversity for agricultural landscapes in the UK. This model can then be used to understand the trade-off between farm management practices on farm economy and the environment. The ability to quantify such trade-offs is critical to our management of the landscape and underpins many sustainability frameworks including the three pillars of sustainability (environmental, economic and social), the UN Sustainable Development goals which includes several targets that relate to agricultural landscapes (Gil et al., 2017), and water-energy-food nexus approaches that aim to consider the use of all of these resources. While tradeoff models exist (e.g. see Sharps et al., 2017) they usually operate at large scales, not accounting for the field or farm scale at which land management decisions are often made. These models are often focussed on land-use options within GIS-based systems, operate on annual time-scales and can be focussed on policy. Our approach, and ultimate aim, is to simulate interactions between the multiple processes that take place in agricultural fields and the farmed landscape with a view to uncovering strategies for development and improvement of agri-environmental systems, beyond the current envelope (Fig. 1). By working on a daily time-step we can simulate the processes and inform the decisions that someone who manages land will have to take.

Here we report the first version of our model that integrates agricultural production, water movement and nutrient flow in a landscape. The model combines aspects of several published models [RothC (Coleman and Jenkinson, 2014), LINTUL (Wolf, 2012), SUCROS (van Laar et al., 1997), and Century (Parton et al., 1994)], but also includes novel factors that have been implemented to capture potential improvements in yield that result from management actions. These include coupling the RothC model to include the dynamics of N and P and responses to changes in bulk-density that result from changes in soil organic matter. We evaluate the model against data on crop growth and nutrient uptake for cereals and for grass, and the integration in space of water and nutrients leaving agricultural fields. We then illustrate how our model can be used to explore trade-offs between production and environment with a scenario based on a wheat crop grown in conditions typical of arable England.

## 2 Methodology

Our intention was to build a model system capable of exploring the multiple interactions between components of a simple landscape and to take into account both within and between field movement of components such as water and nitrate. Nonetheless, because we wished to build a system that can be used on a reasonably large landscape comprising many fields and boundaries, we based our system on simple but adequate descriptions of the processes involved. Here we report on interactions and differences between single or adjacent but joined fields and focus our discussion on productivity and loss of water and nitrogen to water courses and the atmosphere. To do so we describe an integrated model of crop, water and soil processes that runs on a daily time step. We validate this using data from the Broadbalk and Park Grass long-term experiments at Rothamsted Research, in Harpenden, SE England, and spatial interactions are tested on data from the more recently established North Wyke Farm Platform, at Rothamsted Research, near Okehampton, SW England (Orr et al., 2016).

### 2.1 Spatial structure

We impose a grid on the landscape where, dependant on size, each field is represented by one or more grid cells. Soil properties are set in each cell and initial values are given for bulk density, pH and soil water. Within each cell we model crop growth, the dynamics of soil water, total soil organic carbon (TOC), changes in bulk density and nutrient flows on a daily time step. In cases where fields are made up of several cells, water and nutrients can move laterally between cells, as well as vertically though the soil profile. This model structure allows us to explore both temporal and spatial interactions. Cell edges can be designated as ditches (into which water and nutrients may flow), hedgerows or field margins.

### 2.2 Soil water

The soil water model uses a capacity based approach (Addiscott and Whitmore, 1991, Van Ittersum et al., 2003, van Laar et al., 1997). The soil is divided into three layers. This choice is a compromise between capturing the heterogeneity of the soil profile (which would require multiple layers in the simulation) and minimising complexity to enable fast run-times which are important when coupling models with optimisation algorithms over large spatial scales. In our study each layer was initially set to 230 mm. The capacity of each of the soil layers is calculated with van Genuchten (1980) soil water release curves determined using the HYPRES pedo-transfer functions (Wösten et al., 1999). These functions use texture, soil organic matter and bulk density to derive the water release curves. For the topsoil, these release curves are updated daily to take into account changes in bulk density, for example, when farmyard manure (FYM) is added (see Section 2.6).

Infiltrating water fills the soil layers to field capacity (− 10 kPa), and starting from the top layer, excess water drains to the layer below, with water draining from layer 3 becoming drainage. In addition to percolation, water is lost by runoff and evaporation from the soil surface, and transpiration by the growing crop. The water available for crop uptake at any time is equal to the quantity of water stored above wilting point (− 1500 kPa) in the rooted soil profile. A detailed description of the soil water model can be found in van Laar et al. (1997), with our modifications described in Section 2.7. The change in water content in each layer is derived from the balance between inputs from precipitation, and outputs from drainage, runoff, evaporation and transpiration.

Working at the water catchment scale Bell et al. (2007) developed a simple algorithm for estimating the total surface water leaving a sloping (i.e. not uniform in the vertical dimension) region. The storage capacity (*S*) of high zones is reduced in relation to the topographic gradient according to

(1) S=1−g−gmaxSmax

where *S*_*max*_ is the maximum storage capacity, $\overset{-}{g}$ is the average gradient in the cell and *g*_*max*_ is the upper limit on the gradient. By adopting this strategy on a grid cell basis, we increase the flow of water out of each cell compared to that if it were flat. Runoff moves from the highest cell to the lowest by moving between cells with neighbouring boundaries. The proportion of runoff allocated in each direction is determined by the relative magnitude of the downward slopes. Dissolved substances such as nitrate, move in proportion to the water.

### 2.3 Soil total organic carbon, nitrogen and phosphorus

The soil total organic carbon (TOC) model is based on the Rothamsted carbon model, RothC, (Coleman and Jenkinson, 2014). Soil total organic carbon is split into four active compartments and a small amount of inert organic matter (IOM). The four active compartments are Decomposable Plant Material (DPM), Resistant Plant Material (RPM), Microbial Biomass (BIO) and Humified Organic Matter (HUM). Each compartment decomposes by a first-order process with its own rate constant. The IOM compartment is resistant to decomposition. Decomposition of each of the four active pools is modified by rate modifying factors for temperature, moisture and plant retainment. Full details of the model can be found in Coleman and Jenkinson (2014).

The dynamics of the soil organic nitrogen (SON) and soil organic phosphorus (SOP) are modelled in a similar way to the TOC dynamics, both SON and SOP have the same pool structure as the active TOC pools. To determine initial values for each TOC pool, the model is run to equilibrium so that the modelled TOC matches the initial measured TOC. The initial values of each of the SON and SOP pools are then determined using the TOC values, and the C:N and C:P ratios of each pool. The *C* : *N*_Bio_ and *C* : *N*_Hum_ ratios are both fixed at 8.5 (Bradbury et al., 1993), whereas *C* : *N*_DPM_ and *C* : *N*_RPM_ ratios vary over time depending on the carbon inputs to soil from the crop or the addition of organic amendments. The *C* : *P*_Bio_ and *C* : *P*_Hum_ ratios are fixed at 50.0 and 100.0 respectively, like nitrogen the *C* : *P*_DPM_ and *C* : *P*_RPM_ ratios vary over time depending on the carbon inputs to the soil from the crop or the addition of organic amendments.

The N in pool *i* that is mineralised or immobilized is given by

(2) Mi=Δiρi−BiρBio−UiρHum

where Δ_*i*_ is the change in pool *i*  from day *t* to *t* + 1, *B*_*i*_ is the amount of pool *i* transformed to biomass from day *t* to *t* + 1, *U*_*i*_ is the amount of pool *i* transformed to humus from day *t* to *t* + 1, *ρ*_*i*_ is the C:N ratio for pool *i*, and *ρ*_Bio_ and *ρ*_Hum_ are the C:N ratios for the biomass and humus pools respectively. The sum of *M*_i_ across the four pools gives the net mineralisation or immobilization, if the sum of *M*_i_ is negative immobilization occurs and mineral N is removed from the soil, if the sum of *M*_i_ positive is mineralisation occurs and mineral N is added as NH_4_^+^ to the soil. If there is not enough soil mineral N (NO_3_^−^ and NH_4_^+^) on a particular day, then decomposition of TOC does not happen. If there is enough soil mineral N, then N is removed from the NH_4_^+^ pool in preference to NO_3_^−^ pool.

The P mineralisation or immobilization of each SOP pool is calculated in a similar way to the mineralisation N, where in Eq. (2), *ρ*_*i*_ is the C:P ratio for pool *i*, and *ρ*_Bio_ and *ρ*_Hum_ are the C:P ratios for the biomass and humus pools respectively. See Section 2.5 for details on P mineralisation.

### 2.4 Soil mineral nitrogen

In the model, soil mineral N consists of N in ammonium (NH_4_^+^) and nitrate (NO_3_^−^). Inputs of N through atmospheric deposition (*N*_AtDep_) were set to 35 kg N yr^− 1^ (Anon, 1998) for the UK in 1966, decreasing linearly to 20 kg N yr^− 1^ in 2012 (pers. comm. Goulding). Like Sundial (Anon, 1998) it was distributed evenly throughout the year as nitrate. Nitrogen applied as fertilizer enters the NH_4_^+^ or NO_3_^−^ pools depending on the type of fertilizer applied. When organic amendments are added, N enters the soil inorganic nitrogen pools by mineralisation (see Section 2.3).

Rainfall runoff mixes in the model with the water and minerals in the top 20 mm of the soil profile. The amount of mineral nitrogen (NH_4_^+^ and NO_3_^−^) in runoff from the top 20 mm of soil (*N*_Run_) is given by Sharpley (1985)

(3) NRun=NSurfWRunWRun+WSurf

where the surface water (*W*_Surf_) is given by difference in the volumetric water content at saturation and air dried, multiplied by 20 to give the water (mm) in the top 20 mm, *W*_Run_ is the water runoff (mm) and the surface N (*N*_Surf_) is given by

(4) NSurf=20δ1NNH4+NNO3

where *δ*(1) is the depth of the first layer.

Any nitrate in the soil can potentially move down the soil profile with the water. The concentration of NO_3_^−^ in layer *l*, (*γ*_NO3_(*l*)) is given by:

(5) γNO3l=NNO3lWl

where *N*_NO3_(*l*) is the NO_3_^−^ (kg N ha^− 1^) in layer *l*, *l* = 1 … 3, and *W*(*l*) is the water content of layer *l*.

The amount of NO_3_^−^ (kg N d^− 1^) that moves down each layer *l* is given by

(6) FNO3l=max0minNNO3l−1γNO3l−1FWl

where *F*_*W*_(*l*) is the water that flows from layer *l* to layer *l* + 1. The nitrate that moves down from layer 3, *F*_NO3_(3), is N leached out of the profile.

Nitrification is an aerobic process whereby the NH_4_^+^ in the soil is oxidised to form NO_3_^−^ and N_2_O. Our models are based on Milne et al. (2005) and Parton et al. (2001). The rate of nitrification depends on the soil properties, such as water filled pore space *θ*/*θ*_Sat_, soil temperature (T), soil moisture (M), and pH (*S*_pH_). In the model the amount of N_2_O (kg N ha^− 1^ day^− 1^) produced from a given amount of NH_4_^+^ (*N*_NH4 _(*l*)) in layer *l* is given by

(7) NN2Ol=kN2ONNH4lSpHl1−θθSatl

where *k*_N2O_ is a constant that takes the value 0.0001. The amount of nitrate (kg N ha^− 1^ day^− 1^) produced from soil NH_4_^+^ is given by

(8) NNO3l=maxNNH4l−NN2Ol−Nmin1−e−kfTlgMl0

where *N*_min_ is the minimum amount of NH_4_^+^ that must be in the soil for nitrification to occur (we assume *N*_min_ = 0.05), *k* is a constant for nitrification which is set at 0.15, and *f*(*T*(*l*)) and *g*(*M*(*l*)) are functions that describe the effect respectively of temperature and moisture on nitrification, for details see Godwin and Allan (1991).

Denitrification is an anaerobic process whereby the NO_3_^−^ in the soil is reduced to nitrous oxide and nitrogen. The amounts of these gases produced depends on the soil conditions, most notably the nitrate in the soil (*N*_NO3_, kg ha^− 1^), the water filled pore space (*θ*/*θ*_Sat_), and soil temperature (*T*,°C), (Del Grosso et al., 2000, Milne et al., 2011, Nömmik, 1956). We assumed the following simple model to describe N_2_O emissions (kg N ha^− 1^ day^− 1^)

(9) N2Ol=aNNO3lfθ/θSatlgTl

where *a* is a constant. We took the functional forms of *f*(*θ*/*θ*_Sat_) and *g*(*T*(*l*)) from the literature and then fitted the model parameters to data from field experiments from around the UK where nitrate, soil temperature, water filled pore space, and N_2_O emissions (kg N ha^− 1^ day^− 1^) were measured. Similar to other empirical or semi-empirical models, these parameter values can only be assumed to hold for the range of conditions for which they were fitted, and outside of this range further validation would be required. Nitrous oxide is linearly related to nitrate (*N*_NO3_) and we used the function defined by Lark and Milne (2016) to describe the effect of water-filled-pore space on N_2_O emissions. That is

(10) fθ/θSatl=exp−0.6151logθ/θSatl1−θ/θSatl−1.192

where *θ*/*θ*_Sat_(*l*) is the water filled pore space in each layer *l*. Data from Nömmik (1956) suggested that the relationship between temperature and N_2_O emissions should follow a normal distribution with mean 23.65 and standard deviation 5.53. However, data from the Defra project AC0116 (http://www.environmentdata.org/archive/ghgno:676) which we used to relate average temperature to emissions, did not conform to the standard deviation given by Nömmik (1956). Therefore, we assumed the same mean but fitted the standard deviation to our field data. Our fitted model was

(11) N2Ol=0.000735NNO3lfθ/θSatlexp−0.00045Tl−23.652

which we apply in only the top two layers of our model as there is not sufficient biological activity for denitrification to occur in the bottom soil layer.

When water filled pore space increases, the soil becomes more anaerobic and so the amount of N_2_ produced increases. A similar relationship holds for temperature (Nömmik, 1956). We used the following model and fitted the parameters so that our model gave proportions of N_2_O to N_2_ similar to those observed in Colbourn (1988)

(12) N2=0.0052NNO31+e−0.14975T+4.01+e−12.0θ/θSat−0.62

The nitrogen taken up by the crop each day is taken from the nitrate pool with an upper limit of 6 kg N ha^− 1^ day^− 1^ (Semenov et al., 2007).

### 2.5 Soil mineral phosphorus

In the model, mineral phosphorus is split into two pools: available P (which includes phosphorus in soil solution and loosely adsorbed to the clay surface) and non-available P. Eighty percent of the fertilizer P enters the available P pool and the remaining 20% enters the non-available P pool (Wolf et al., 1987).

Similar to the N model, a proportion of the available P contained in the top 20 mm of soil can be lost through runoff

(13) PRun=PSurfWRunWRun+WSurf

where the surface P (*P*_Surf_) is given by

(14) PSurf=20δ1PM

where *P*_*M*_ is the mobile (dissolved and particulate) P which we assume to be 10% of *P*_AV._ We set solution P to 1% of the available P (pers. comm. Paul Poulton). This can potentially be leached when water flows down the profile.

The soil organic P that is mineralised is added to the available P pool. Mineral P may also be immobilized, in which case it is taken from the available P pool first and then from the non-available P pool.

Available P (*P*_Av_) is converted to non-available P (*P*_NonAv_) by reversible processes which reduce its extractability. In the model, the P content for each soil layer (available and non-available P), which we define *P*_Tot_, is calculated in mg kg^− 1^ soil. The release to fixation variable, *V*(*l*), for layer *l* is given by

(15) Vl=αbPTotl+βbPTotl,PTotl>βa−βbαb−αaαaPTotl+βaPTotl,PTotl≤βa−βbαb−αa

were *α*_*b*_ and *β*_*b*_ are the slope and intercept, of value 0.113 and − 49.3 respectively, for the linear relationship between *P*_Av_ and *P*_Tot_. For small values of *P*_Tot_, an alternative set of coefficients *α*_*a*_ and *β*_*a*_, of value 0.0201 and − 5.1, are used (see Supplementary Fig. 1). The ratio of release to fixation is given by

(16) RRFl=Vl1.0−Vl

The transfer of P from the non-available to the available pool *P*_NA → Av_, and the reverse transfer *P*_Av → NA_ in layer *l* on day *t* + 1 are given by

(17) PAV→NA=λPAvltfpHSpHl

(18) PNA→Av=λPNonAvlTRRFlfpHSpHl

and so

(19) PNonAvlt+1=PNonAvlt+PAv→NA−PNA→Av

(20) PAvlt+1=PAvlt−PAv→NA+PNA→Av

The constant *λ* determines the rate of re-equilibration between *P*_Av_ and *P*_NonAv_ following the addition of mineral P, and is set to 0.01 giving a half-life of approximately 65 days. The values of coefficients, *α*, *β* and *λ* were established for a silty clay loam soil at Rothamsted. The rate modifying function *f*_pH_ linearly increases from 0.0 to 1.0 as pH increases from 0 to 7, and then linearly decreases back to zero as pH increases from 7 to 14. The P required by the crop is taken from the available P pool, up to a limit of 2 kg P ha^− 1^ day^− 1^.

### 2.6 Bulk density

To take into account changes in depth caused by changes in bulk density as a result of, for example, the addition of FYM, we used the Rawls (1983) nomogram to estimate bulk density in relation to sand, clay and organic carbon contents of soil. The depth of the topsoil is modified to reflect the change in bulk density (changes in depth and bulk density only occur in the top soil). Because of the changes in depth and bulk density in the top soil, we modify water properties, such as the water content at saturation, field capacity, and wilting point, daily (see Section 2.2). Modelling bulk density dynamically in this way has been described previously by Whitmore et al. (2011).

### 2.7 Crop model

Our crop model is a generic plant growth model, which uses a light use efficiency (LUE, g dry matter MJ^− 1^) based approach to calculate the biomass production (Monteith, 1990, Monteith and Moss, 1977). The rate of biomass (*B*_crop_) produced each day is given by

(21) dBcropdt=QεWrfNNIPNI

where *Q* is the intercepted PAR (MJ PAR m^− 2^ surface area) which depends on the solar radiation and canopy leaf area, *ε* is the crop specific LUE, which for grass, changes with development stage see Schapendonk et al. (1998), *W*_*rf*_ is the transpiration reduction factor, *N*_*NI*_ and *P*_*NI*_ are the nitrogen and phosphorus nutrition indices, which range from zero to one. For grass, LUE is reduced for higher radiation levels (Schapendonk et al., 1998). In our model LUE is reduced by a factor *R*_*LUE*_ which decreases from 1.0 to 0.33 when radiation increases from 10 to 40 MJ m^− 2^ d^− 1^. Schapendonk et al. (1998) also modified LUE, by the temperature factor *T*_LUE_, which in this study increases linearly from 0.0 to 1 between 6.0 and 9.0 °C. The biomass formed is partitioned between roots, stem, leaves and storage organs based on the development stage (DVS) (Boons-Prins et al., 1993, Wolf, 2012).

The transpiration reduction factor (*W*_rf_) is defined as the ratio of actual transpiration (mm day^− 1^) to potential transpiration (mm day^− 1^) and is calculated

(22) Wrf=∑l=13ATranlPTran

where *P*_*Tran*_ is the daily potential transpiration which is calculated as in Lintel (Wolf, 2012).

The amount of the actual transpiration coming out of layer (l) is given by

(23) ATranl=PTranlWSl2FRLlWS1FRL1+WS2FRL2+WS3FRL3

Here *F*_RL_ is the fraction of root in each layer and *W*_*S*_ is the impact of water content on the water stress function. This follows the approach of Li et al. (2001). This impact of water content is based on the method described in Feddes et al. (1976) given by

(24) WS=θs−θθs−θa,forθ>θa1forθa≥θ>θdθ−θwθd−θw,forθd≥θ>θw

where *θ* is the volumetric water content, *θ*_*s*_ is the water content at saturation, *θ_a_* is the water content at − 5 kPa, *θ_d_* is the water content at − 40 kPa, and *θ*_*w*_ is the water content at wilting point (− 1500 kPa). Water stress affects grass less than arable crops (per comms J. Storkey). In simulations, when the soil is saturated grass does not suffer water stress. When the volumetric water content falls below *θ_d_* = − 40 kPa the water stress factor *W*_*S*_ decreases linearly between *θ_d_* and *θ_w_* to 0.4.

The proportion of root (*F*_RL_) in each layer *l* is given by

(25) FRLl=RLenlRLen1+RLen2+RLen3

where *R*_Len_ is the root length per unit area (mm mm^− 2^).

The root depth (*d*_root_) increases by 12.0 mm per day to a maximum root depth which depends on the crop being modelled. The root length per unit area within each layer, calculated according to an adaptation of the method of Gerwitz and Page (1974), is given by

(26) RLenl=−R0ae−aZ2l−e−aZ1l

where *R*_0_ is the root length density at the soil surface (mm mm^− 3^) the value of which is non-essential to the model as it cancels out in Eq. (25), *z*_1_(*l*) and *z*_2_(*l*) are the upper and lower horizon depth (mm) of layer *l*, and *a* is given by

(27) a=−ln1−Frdroot

where *F*_*r*_ is the fraction (arbitrarily defined as 0.98) of the root length that is present above *d*_root_.

The uptake of plant nutrient (N and P) is determined by the crop demand and the supply of these nutrients by soil. The total nutrient demand of the crop is the sum of the nutrient demand from its individual organs (i.e. roots, stems and leaves excluding storage organs, for which nutrient demand is met by translocation from the other organs). Nutrient demand of the individual organs is calculated as the difference between maximum and actual organ nutrient contents. The maximum nutrient content is defined as a function of canopy development stage. The total nutrient uptake of the crop takes place before anthesis. Sub-optimal nutrient availability in the soil leads to nutrient stress in the crop. A detailed description of crop nitrogen dynamics is reported by Shibu et al. (2010) and P dynamics follows N in a similar way.

Nitrogen stress in the plant growth model is expressed as nitrogen nutrition index (*N*_NI_) and is calculated by:

(28) NNI=max0min1Nleaf+Nstem−NResΩleaf+ΩstemΩleafNMaxPropleaf+ΩstemNMaxPropstem−NResΩleaf+Ωstem

where *N*_leaf_ and *N*_stem_ are the *N* in the leaf and stem respectively, *Ω*_leaf_ and *Ω*_stem_ are the weights of the leaf and stem respectively, *N*_MaxPropleaf_ and *N*_MaxPropstem_ are the maximum proportion of N in the leaf and stem respectively. The residual N (*N*_Res_) is the fraction of N which is part of the cell structure and was fixed at 0.004 for wheat (Wolf, 2012) and 0.01 for grass (Bouman et al., 1996). For wheat, the maximum N in the leaf is given by:

(29) NMaxPropleaf=0.046exp−1.7D+0.014

where *D* is the development stage of the crop which is calculated using thermal time modified by a vernalisation factor and the photosensitivity of the crop (see Wolf, 2012, and references therein). For grass we set *N*_MaxPropleaf_ to 0.0425. The maximum N in the stem is given by *N*_MaxPropStem_ = 0.5 *N*_MaxPropLeaf_, (see Wolf, 2012).

The phosphorus nutrition index (*P*_NI_) is calculated by:

(30) PNI=max0min1Pleaf+Pstem−ΩleafPResLeaf+ΩstemPResStemΩleafPMaxPropleaf+ΩstemPMaxPropstem−PResΩleafPResLeaf+ΩstemPResStem

where *P*_leaf_ and *P*_Stem_ are the P in the leaf and stem respectively, and *P*_MaxPropleaf_ and *P*_MaxPropstem_ are the maximum proportion of P in the leaf and stem respectively. For wheat the residual P in the leaf is *P*_ResLeaf_ = 0.0003 and in the stem *P*_ResStem_ = 0.00018. For grass both *P*_ResLeaf_ and *P*_ResStem_ are set to 0.001 (Wolf et al., 1987). For wheat the maximum P in the leaf reduces with development stage. From development stages 0 to 0.7 it reduces linearly from 0.0066 to 0.0036 and then from 0.0036 to 0.0009 from development stage 0.7 to 1, after which it holds the value of 0.0009. For grass the maximum P in the leaf is fixed at 0.0035 (Bouman et al., 1996).

Processes leading to the aboveground litter formation and carbon turnover below ground are similar for both crops and grass but their rates are different. We assume that 50% of the dead leaves become litter on a daily basis and the remainder is left on the stem. The rate at which the roots die is a function of growth stage. In the case of crops, the root death happens towards the latter part of the growing season (DVS > 1.5) at a rate of 0.02 per day. In the case of grass, once the root system has been established (3–6 months after sowing, DVS = 0.01), root death becomes continuous at a rate of 0.01 per day. The root exudates are considered to be a part of root death, so are not modelled separately. The leaf death rate is a function of heat stress, nitrogen stress and shading as described in Schapendonk et al. (1998). All C, N, and P from dead roots and litter is returned to the soil.

The grass model differs somewhat from the crop model as grass has indeterminate growth and is not allowed to flower (so always has a DVS < 1.0) as it can be cut or grazed in the model (unlike the crop which completes its life cycle in a given growing season). Grass is a perennial crop that grows for one or more seasons before being reseeded. Cut grass and grazed grass is removed from the modelled system. The amount removed is such that the remaining biomass cannot fall below 50 g m^− 2^. Livestock deposit nutrients into the system as manure. When animals are on the field, we set the deposition of C and N for each animal type based on data from Cottrill and Smith (2007), for each beef animal this was 4.03 kg C of manure per day containing 0.22 kg N, for each dairy cow this was 6.45 kg C per day containing 0.35 kg N, and for each sheep this was 0.45 kg C per day as fresh deposit, containing 0.02 kg N per day. These rates are multiplied by the stocking rate to give the rate of deposit per hectare.

### 2.8 Data requirements

For each layer of the soil, the model requires initial values for soil depth, clay, silt, TOC, bulk density, available P, non-available P, soil NH_4_, soil NO_3_, soil pH. Initial values for elevation and latitude are also needed. The model runs with a daily time-step and so for each simulated day weather data (minimum and maximum temperature, rainfall, radiation, vapour pressure and windspeed) are needed. For each season and where relevant to the crop, sowing dates, fertilizer application timing, type and dose and dates when the grass is cut are required.

### 2.9 Case studies

To test our model, we used data from two long-term agricultural experiments and one more recent grass-livestock experiment. These were: The Broadbalk wheat experiment, and the Park Grass permanent grassland experiment at Rothamsted Research, Hertfordshire, UK (51.8° N, 0.37° W), and the more recent North Wyke farm platform at Rothamsted Research, near Okehampton, UK (50.77° N, 3.92° W), which has spatially integrated data from livestock-bearing grassland in a sloping terrain. We used a suite of statistical metrics (including the mean, standard deviation, root mean square error, and sample correlation coefficient, r) to quantify the performance of our model (see Smith et al., 1997).

#### 2.9.1 Broadbalk

The Broadbalk wheat experiment has been running since 1843, and wheat has been sown and harvested on all or part of the experiment every year since then. The original aim of the experiment was to test the effects of various combinations of inorganic fertilizers and organic manures on the yield of winter wheat. The experiment was divided into different strips given a range of fertilizer applications, which extended the whole length of the field. In 1926 the experiment was divided into five Sections, crossing the fertilizer treatments at right angles, where each section was bare fallowed one year in five to control weeds. In 1968 the experiment was further divided into 10 Sections, so that the yield of wheat grown continuously could be compared with that grown in rotation after a two-year break. The plots receive management consistent with standard practice for the time. The soil is clay loam to silty clay loam, predominately Batcombe series (Avery and Catt, 1995), FAO classification: Chromic Luvisol (or Alisol), U.S. Soil Taxonomy: Aquic (or Typic) Paleudalf. The site is thought to have been in arable cropping for many centuries before the start of the experiment. Further details are available from http://www.era.rothamsted.ac.uk/Broadbalk

The plots from the continuous wheat sections (Sections 1 and 9), selected for this study, receive a range of fertilizer and FYM applications (see Table 1). Wheat has been grown every year on these Sections, since 1966. Modern, short-strawed high yielding varieties were introduced in the 1967–1968 season and it is from this date that we test the model. Most of the data are available from the electronic Rothamsted Archive (e-RA http://www.era.rothamsted.ac.uk). Periodic measurements of TOC were made on all plots (Watts et al., 2006; Pers. comm. P. Poulton for later data), measurements of volumetric water content on plot 8 in 2007 (Pers. Comm, C. Watts) and measurements and estimates of N leaching were made between 1990 and 1998 (Goulding et al., 2000). Grain N was measured 1968–2012, and grain P from 1968 to 2011 (except 1976–1985), Section 1 only.

**Table 1:** The fertilizer and manure treatments applied annually to the Broadbalk experiment plots used in the simulations.

Treatments					
Plot	Up to 1967	1968–1984	1985–2000	2001–2004	2005–2012
3	Nil	Nil	Nil	Nil	Nil
5	P K Na Mg	P K Na Mg	P K Mg	K Mg	K Mg
6	48 N P K Na Mg	48 N P K Na Mg	48 N P K Mg	48 N K Mg	48 N K Mg
7	96 N P K Na Mg	96 N P K Na Mg	96 N P K Mg	96 N K Mg	96 N K Mg
8	144 N P K Na Mg	144 N P K Na Mg	144 N P K Mg	144 N K Mg	144 N K Mg
9	48 N* P K Na Mg	192 N P K Na Mg	192 N P K Mg	192 N K Mg	192 N K Mg
15	96 N P K Na Mg	144 N P K Na Mg	240 N P K Mg	240 N K Mg	240 N K Mg
16	96 N* P K Na Mg	96 N P K Na Mg	288 N P K Mg	288 N K Mg	288 N K Mg
2.1	FYM since 1885	FYM 96 N	FYM 96 N	FYM 96 N	FYM 144 N
2.2	FYM	FYM	FYM	FYM	FYM

The values of N are in kg N ha^− 1^, applied as ammonium sulphate 1843–1967, as calcium ammonium nitrate between 1968 and 1985, and as ammonium nitrate thereafter. Treatments with * were applied as sodium nitrate. Farmyard manure (FYM) was applied at 35 t ha^− 1^ fresh weight, and contains approximately 250 kg N ha^− 1^. Other elements were applied at 35 kg P ha^− 1^, 90 kg K ha^− 1^, 16 kg Na ha^− 1^ until 1973 and 12 kg Mg ha^− 1^ respectively. P has not been applied since 2001, due to high levels of plant available P in the soil. For more details see http://www.era.rothamsted.ac.uk/Broadbalk

We ran the model to simulate the plots listed in Table 1 using weather data from the Rothamsted meteorological station from 1966 to 2012. Comparisons were made between measured and simulated values of crop yield, content of N and P in the grain, TOC, volumetric water content and nitrate leaching.

#### 2.9.2 Park Grass

The Park Grass experiment is the oldest experiment on permanent grassland in the world. Started by Lawes and Gilbert in 1856, its original purpose was to investigate ways of improving the yield of hay by the application of inorganic fertilizers and organic manure. Within three years it became clear that these treatments were having a dramatic effect on the species composition of what had been a uniform sward. The continuing effects of the original treatments on species diversity and on soil function, together with later tests of liming and interactions with atmospheric inputs and climate change (Storkey et al., 2015), has meant that Park Grass has become increasingly important to ecologists, environmentalists and soil scientists. The soil is silty clay loam, predominately Hook series, with areas more typical of the Batcombe series (Avery and Catt, 1995), FAO Classification: Chromic Luvisol (or Alisol), U.S. Soil Taxonomy: Aquic (or Typic) Paleudalf. The site is known to have been in permanent pasture for at least 100 years before the start of the experiment. For further details see http://www.era.rothamsted.ac.uk/Park

The plots are cut in mid-June, and made into hay. A second cut is usually taken in the autumn, except in a few years, when there was insufficient herbage to sample. Since 1960, yields have been estimated from strips cut with a forage harvester. The remainder of the plot is still mown and made into hay, continuing earlier management. For the second cut, the whole of each plot is cut with a forage harvester. The experiment is never cultivated, and the site was in permanent grassland for at least 100 years before the experiment began. Further details are available from http://www.era.rothamsted.ac.uk/Park

Here we simulated two plots, Plot 3a and 14/2a, with contrasting fertilizer treatments. Plot 3a has received no inorganic fertilizer or manure since 1856. Plot 14/2a has received 96 kg N ha^− 1^ in the spring, and 35 kg P in the autumn each year since 1858, plus K, Na and Mg. In 1965 the plots were divided into four subplots, given different amounts of chalk to maintain soil at pHs of 7, 6 and 5 (sub-plots a, b and c, respectively). The fourth sub plot (d) receives no chalk. We have selected sub-plot ‘a’ for this simulation, with a pH of 7. We use yield data from 1966 to 2012, with two cuts each year except in 2003, when no second cut was taken, with weather data from the Rothamsted meteorological station.

We chose Plot 14/2a over the other N fertilizer plots because N is applied as sodium nitrate, whereas in most other plots N is applied as ammonium sulphate, which has an acidifying effect on the soil and so a dramatic effect on species composition and the decomposition of soil organic matter (see http://www.era.rothamsted.ac.uk/Park).

#### 2.9.3 The North Wyke Farm Platform

The North Wyke Farm Platform, near Okehampton, SW England was established as a UK National Capability for collaborative research, training and knowledge exchange in agro-environmental sciences related to beef and sheep production in lowland grasslands (Orr et al., 2016). The soils on the farm platform are predominately Halstow series, (Pelo-stagnogley soils, Avery, 1980), FAO Classification: Stagni-vertic cambisol, U.S. Soil Taxonomy: Typic haplaquept. For more details see Harrod and Hogan (2008). A system based on permanent pasture was implemented on three 21-ha farmlets to obtain baseline data on hydrology, nutrient cycling and productivity for two years. Since then, two of the farmlets have been modified by either (i) planned reseeding with grasses that have been bred for enhanced sugar content or deep-rooting traits or (ii) sowing grass and legume mixtures to reduce nitrogen fertilizer inputs. The third farmlet continued under permanent pasture. The quantities of nutrients that enter, cycle within and leave the farmlets are recorded using sensor technologies alongside more traditional field study methods. Here we simulated the water and nutrient flows from October 2012 to 25th December 2013 from catchment 4 (Golden Rove) and catchment 5 (Orchard Dean), two of the un-modified permanent grassland catchments, that had contrasting topologies. The North Wyke data that we used for this study are available from http://www.rothamsted.ac.uk/farmplatform.

### 2.10 Trade offs

We coupled the simulation model with an optimisation algorithm to determine Pareto optimal fronts between multiple objectives defined in terms of outputs from the model. The optimised Pareto fronts describe the trade-offs between objective variables such as yield and nitrate leaching. To illustrate how these can be identified, we used the fertilizer application time and amount as two management variables that the optimisation algorithm could vary in order to affect three objectives: the yield of a wheat crop, nitrate leaching and N_2_O emissions. Simulations used the soil properties and weather data from plot 9 of the Broadbalk experiment for the years 1968–1978. For this period the mean measured yield was 5.4 t ha^− 1^ at 85% dry matter.

**Fig. 2:**
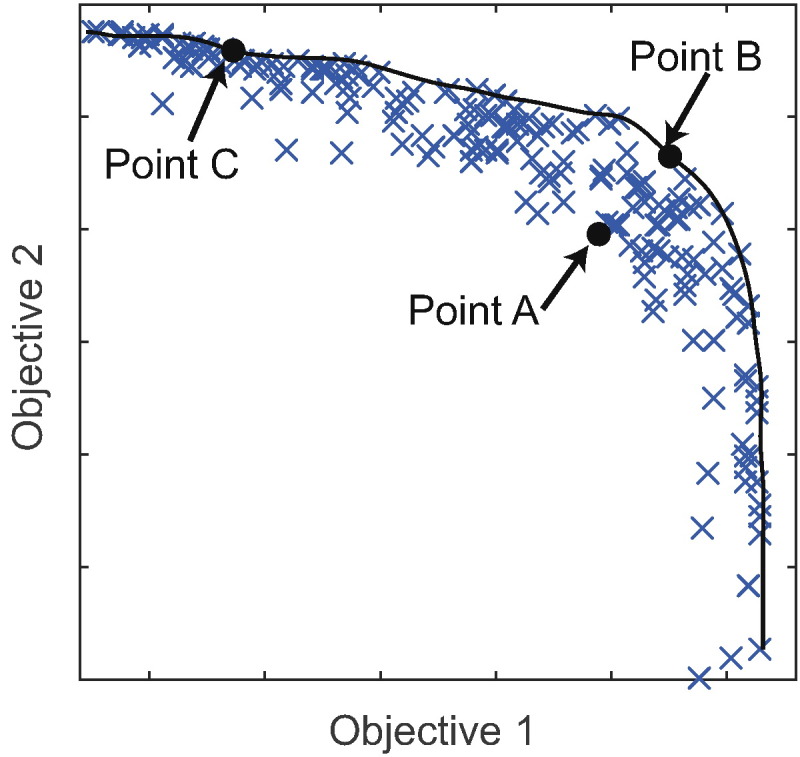
Example of how a Pareto front is identified from a number of points simulated by the model with the aim to improve multiple objectives (1 & 2) simultaneously. Point B is selected over point A because B scores better for both objectives. It can be seen that neither of points B or C dominates the other, because point B does better at objective 1 whilst point C improves on objective 2. Consequently, both are retained. The Pareto front (line) can be identified by connecting together all of the non-dominated points.

Initially the algorithm, which combines non-dominated sorting (Deb et al., 2002) with differential evolution (Storn and Price, 1997), randomly selects a number of possible management variables, implements these management options in the simulation model and records the effect on each of the multiple objectives. Non-dominated sorting then identifies the management options that result in the ‘best’ objectives, i.e. those that are non-dominated. A point is said to be dominated by another if it is worse for every single objective. For example, if we aim to maximise two objectives, point A (Fig. 2) is dominated by point B because the value of both objectives is greater at B than A. Points B and C, however, are both non-dominated with respect to one another because whilst objective 1 is higher for B, objective 2 is higher for C. The non-dominated sorting algorithm performs a series of pairwise comparisons in order to identify all of the management options that lead to non-dominated sets of objectives. The differential evolution algorithm then combines aspects of the management options that led to non-dominated objectives to identify new management options that could potentially perform even better. The process is iterated in directions that the differential evolution algorithm suggests will be an improvement, until the results converge and produce a similar Pareto front with each iteration.

## 3 Results

### 3.1 Broadbalk

**Fig. 3:**
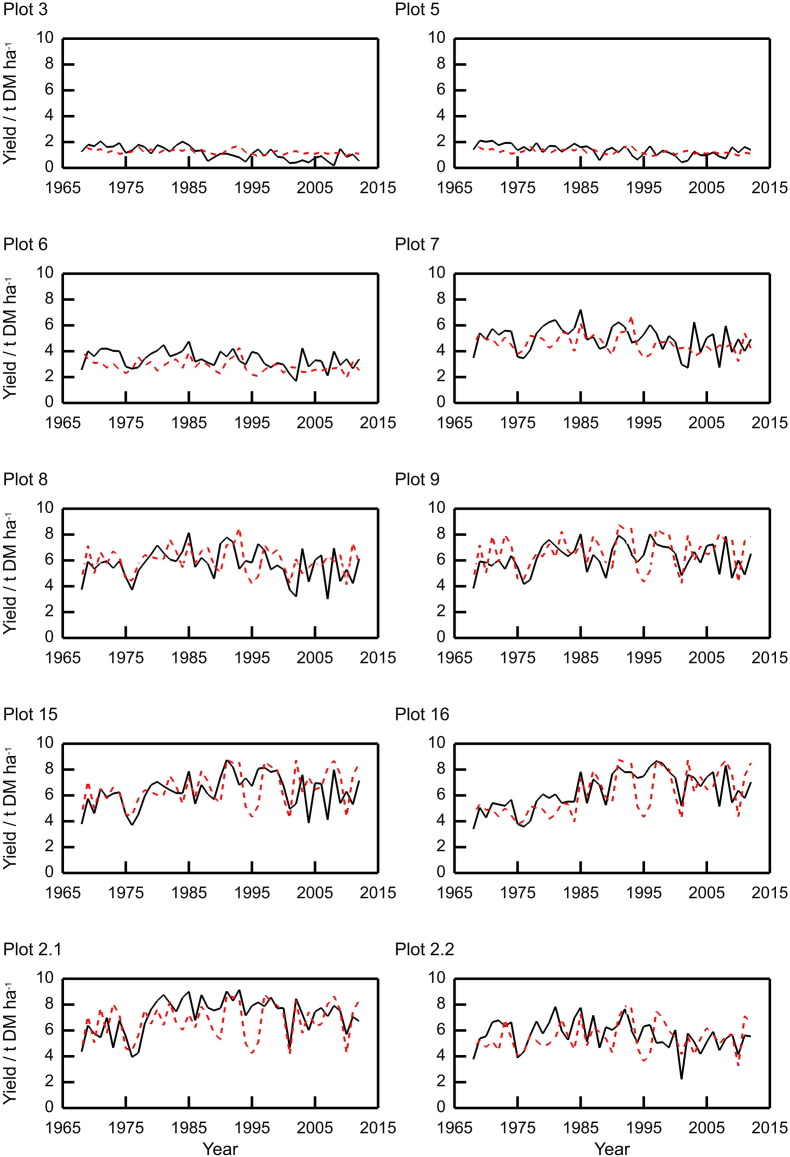
Measured (black lines) and modelled (red dashed lines) grain yields for ten plots from the Broadbalk long-term wheat experiment, 1968–2012, continuous wheat (Sections 1 and 9). The measured values were averaged over Sections 1 and 9 (see 2.9.1).

The simulated and measured grain yields for the plots listed in Table 1 are shown in Fig. 3. The model captures the differences between the plots well and this is quantified by the overall correlation between modelled and measured (Pearson correlation, *r* = 0.86). The plot means for the modelled and measured yields are similar, as are the variances, although the variance for the modelled yield in plots with little fertilizer N applied are smaller than the observed (Table 2). The model reflects the year-to-year fluctuations in yield, although notably under-predicts the 1995 yield from the plots with larger N applications (9, 15, 16, 2.1 and 2.2).

**Table 2:** Summary statistics for measured and simulated grain yields (at 85% dry matter), 19,68-2,012 for the Broadbalk wheat experiment. The measured values for yield in each year were averaged over Sections 1 and 9 (see 2.9.1).

Plot no.	Measured		Simulated			
	Mean t ha^− 1^	Standard deviation/t ha^− 1^	Mean t ha^− 1^	Standard deviation/t ha^− 1^	RMSE (%)	Correlation
3	1.16	0.5	1.26	0.22	42.56	0.28
5	1.37	0.42	1.27	0.22	33.41	0.16
6	3.4	0.67	2.86	0.51	28.61	0.07
7	4.99	1.02	4.62	0.7	24.03	0.16
8	5.71	1.18	6	1.01	24.27	0.25
9	6.23	1.06	6.66	1.32	23.01	0.36
15	6.32	1.28	6.58	1.37	23.29	0.4
16	6.34	1.41	6.12	1.64	20.88	0.64
2.1	7.16	1.35	6.75	1.41	20.28	0.49
2.2	5.67	1.12	5.49	1.13	22.84	0.35

**Fig. 4:**
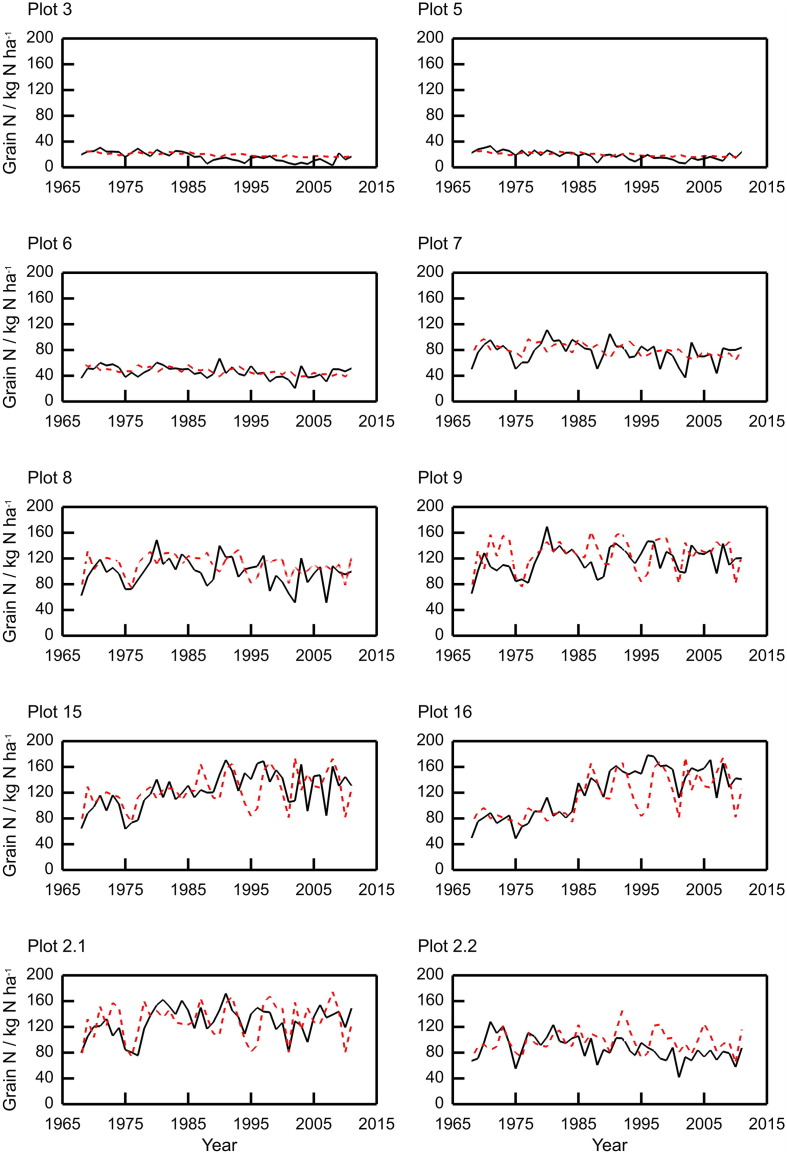
Measured (black lines) and modelled (red dashed lines) grain N content for ten plots from the Broadbalk long-term wheat experiment, 1968–2012, continuous wheat. The measured values were from Section 1 only (see 2.9.1).

**Fig. 5:**
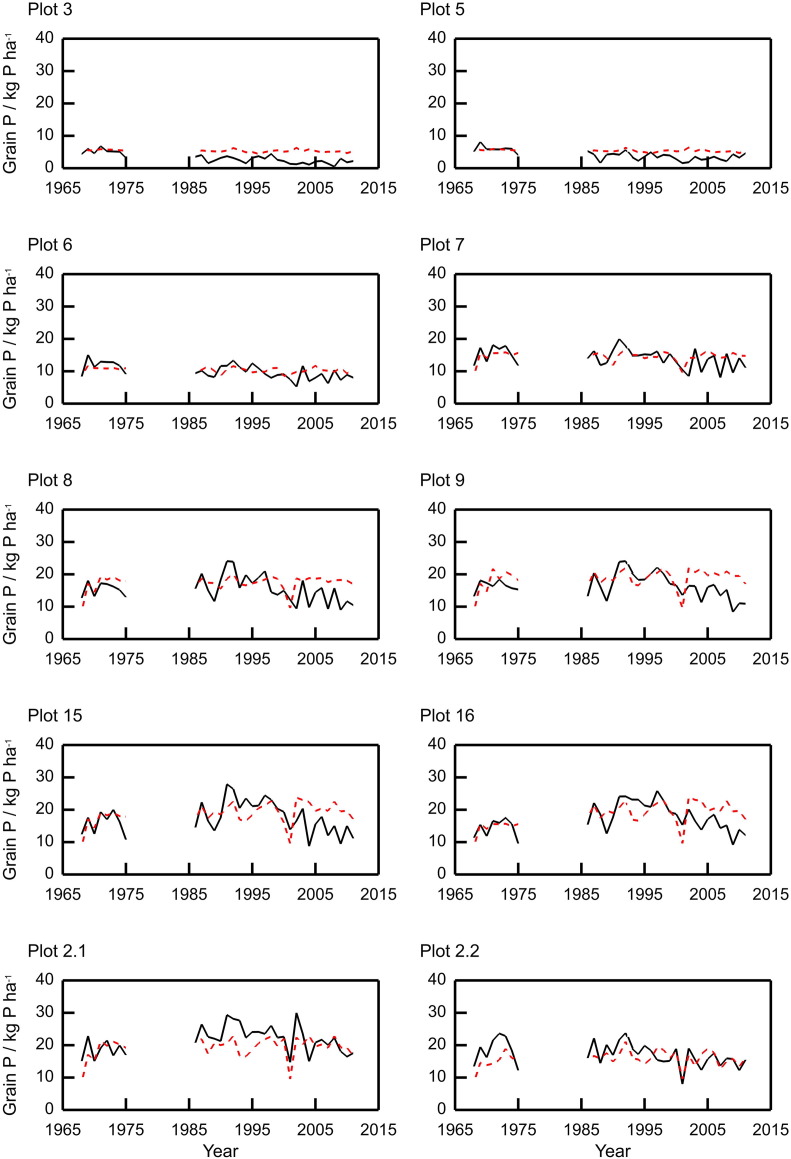
Measured (black lines) and modelled (red dashed lines) grain P content for ten plots from the Broadbalk long-term wheat experiment (1968–1975 and 1986–2011), continuous wheat. The measured values were from Section 1 only (see 2.9.1).

**Fig. 6:**
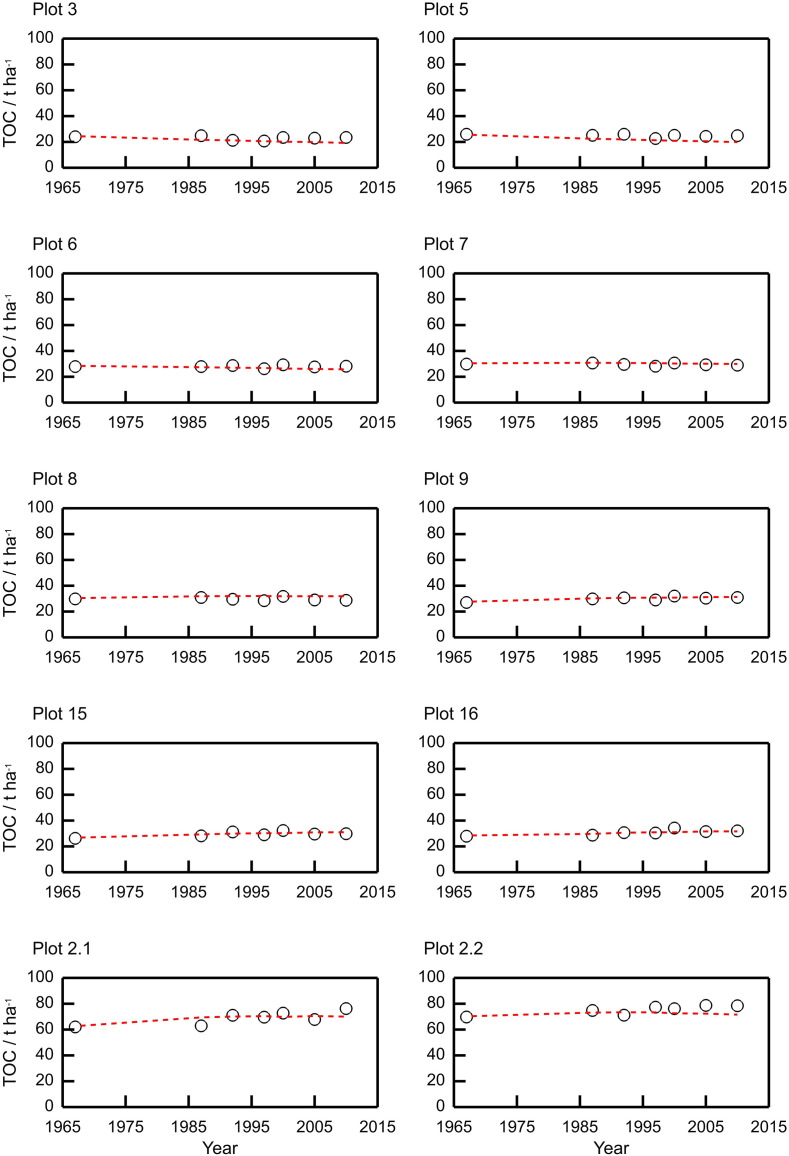
Measured (black circles) and modelled (red dashed lines) soil total organic carbon (TOC) for ten plots from the Broadbalk long-term wheat experiment. The measured values were averaged over Sections 1 and 9.

**Fig. 7:**
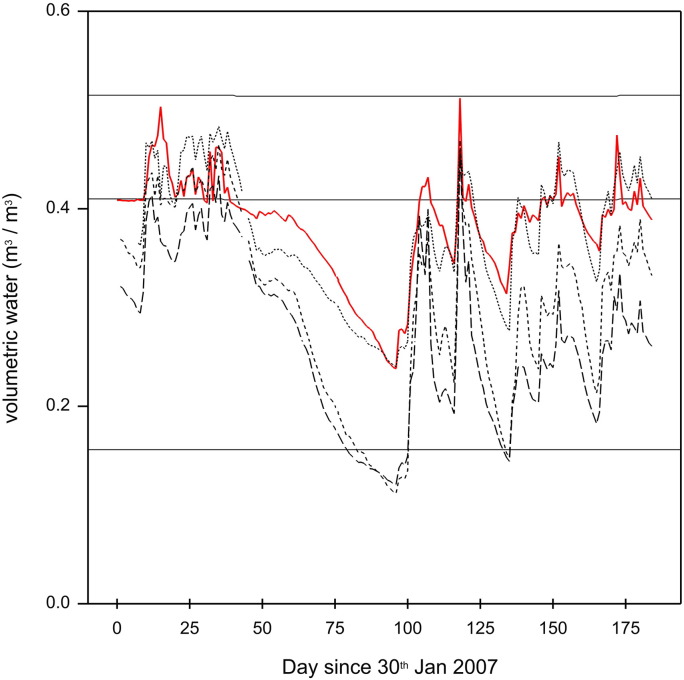
Measured for three replicates (black dashed lines) and modelled (red line) volumetric water content in soil from plot 8 of the Broadbalk experiment.

The model replicates the plot-to-plot and year-to-year variation in grain N, grain P and TOC (see Fig. 4, Fig. 5, Fig. 6, and Table 3, Table 4, Table 5), although we note that year-to-year variation in TOC is minimal. The correlations across all plots between modelled and measured grain N, grain P, and TOC are 0.88, 0.84 and 0.99 respectively. The model reproduces the pattern in the variation of volumetric water content for plot 8, following one of the observed realisations closely (Fig. 7). Note that measurements with such probes are sometimes biased towards drier measurements because instrument range is short and if contact is lost between the access tube and soil then the soil can appear drier than it actually is.

**Table 3:** Summary statistics for measured and simulated grain N content, 1968–2012, for the Broadbalk wheat experiment. The measured values for grain N were from Section 1 only (see 2.9.1).

Plot no.	Measured		Simulated			
	Mean kg N ha^− 1^	Standard deviation/ kg N ha^− 1^	Mean kg N ha^− 1^	Standard deviation/ kg N ha^− 1^	RMSE (%)	Correlation
3	16.33	7.23	19.76	3.1	42.54	0.57
5	18.48	6.36	20.07	3.15	31.58	0.47
6	46	9.12	47.69	5.79	23.42	0.03
7	76.69	16.21	80.44	9.03	23.58	0.11
8	99.03	21.31	110.25	15.84	25.44	0.29
9	117.91	20.91	126.27	23.92	23.32	0.32
15	122.99	28.03	124.05	25.96	25.17	0.35
16	121.34	37.12	113.62	32.82	22.98	0.71
2.1	128.08	23.56	129.28	27.52	21.27	0.44
2.2	86.83	18.58	98.09	17.27	27.04	0.34

**Table 4:** Summary statistics for measured and simulated P in the grain, 1968–1975 and 1986–2011 for the Broadbalk wheat experiment. The measured values for grain P were from Section 1 only (see 2.9.1).

Plot no.	Measured		Simulated			
	Mean kg P ha^− 1^	Standard deviation/ kg P ha^− 1^	Mean kg P ha^− 1^	Standard deviation/ kg P ha^− 1^	RMSE (%)	Correlation
3	3.04	1.49	5.36	0.43	89.47	0.29
5	4.05	1.47	5.4	0.43	48.33	0.26
6	9.89	2.23	10.35	0.86	22.5	0.26
7	14.11	2.89	14.58	1.66	20.73	0.29
8	15.38	3.85	17.31	2.38	29.01	0.23
9	16.49	3.59	18.64	3.06	27.8	0.26
15	17.47	4.73	18.77	3.27	28.34	0.33
16	17.33	4.3	18.42	3.57	25.47	0.42
2.1	21.49	4.09	19.36	3.26	20.42	0.47
2.2	17.14	3.57	15.8	2.5	20.24	0.49

**Table 5:** Summary statistics for measured and simulated total soil organic carbon (TOC), measured between 1967 and 2012 for the Broadbalk wheat experiment. The measured values for TOC were averaged over Sections 1 and 9 (see 2.9.1).

Plot no.	Measured		Simulated			
	Mean t C ha^− 1^	Standard deviation/ t C ha^− 1^	Mean t C ha^− 1^	Standard deviation/ t C ha^− 1^	RMSE (%)	Correlation
3	22.95	1.37	21.01	1.6	11.51	0.28
5	24.84	1.05	21.78	1.76	13.83	0.47
6	27.96	0.88	26.82	0.84	6.07	− 0.08
7	29.57	0.83	30.39	0.3	3.88	0.24
8	29.73	1.12	31.69	0.53	7.88	− 0.1
9	29.97	1.47	30.36	1.18	3.11	0.82
15	29.45	1.84	29.74	1.33	4.42	0.72
16	30.75	1.96	30.55	1.06	4.3	0.78
2.1	68.9	4.76	68.97	2.61	5.46	0.62
2.2	75.18	3.27	72.36	1.06	5.61	0.28

**Fig. 8:**
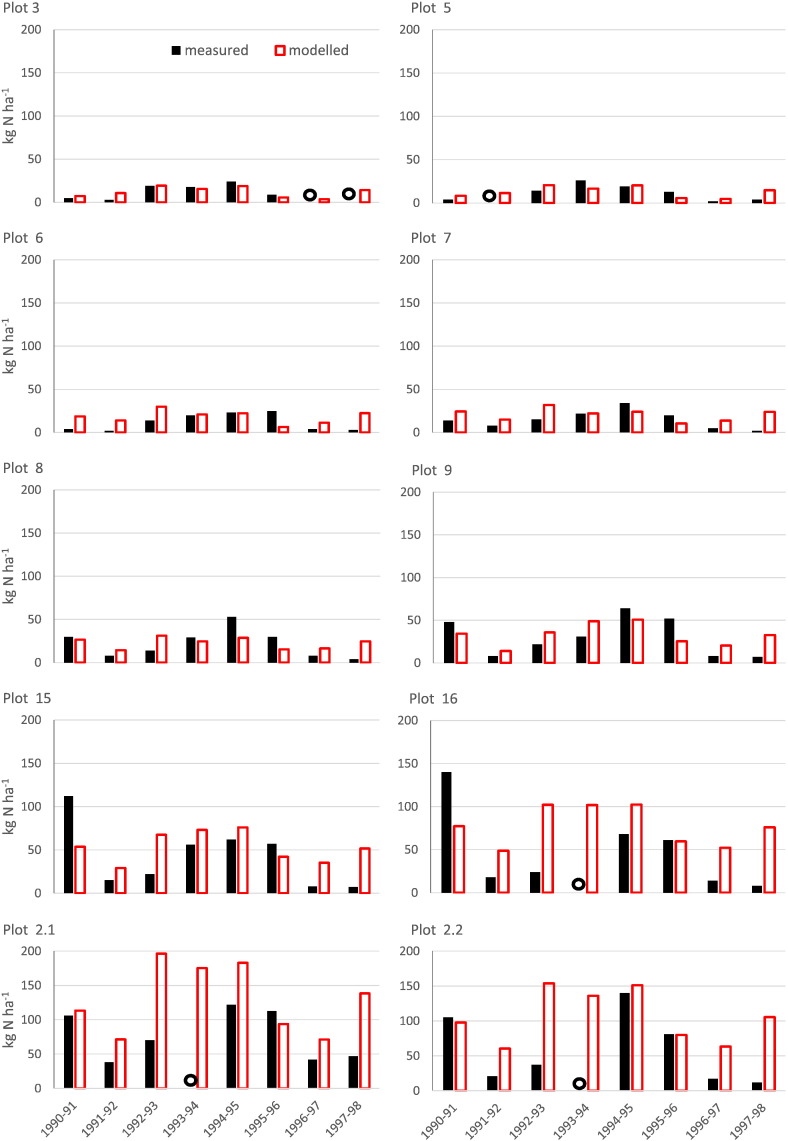
Estimated and modelled N leached from study plots on the Broadbalk wheat experiment 1990–1998. Measurements are from Section 9 only. The black open circle indicates that no measurement was taken.

The measured (Goulding et al., 2000) and modelled N leached for each plot are shown in Fig. 8. The model predictions match the N leached from the mineral fertilized plots reasonably well, although the model consistently overestimates N leached from plots receiving the most N (plots 15 and 16 and the FYM plots 2.1 and 2.2) and in the driest years (1991/2, 1996/7 and 1997/8). The variances for measured leaching are larger than the modelled for all but plot 2.1 (Table 6). Note that measurements were not determined for every plot in every year.

**Table 6:** Summary statistics for measured and simulated nitrate leached (kg N ha^− 1^ y^− 1^) between 1990 and 1998, Broadbalk wheat experiment. Measurements are from Section 9 only.

Plot no.	Measured		Simulated			
	Mean kg N ha^− 1^ y^− 1^	Standard deviation/ kg N ha^− 1^ y^− 1^	Mean kg N ha^− 1^ y^− 1^	Standard deviation/ kg N ha^− 1^ y^− 1^	RMSE (%)	Correlation
3	13.00	8.51	11.94	5.99	33.29	0.85
5	11.71	8.92	12.86	6.21	58.56	0.60
6	11.88	9.76	18.24	7.38	111.01	− 0.02
7	15.00	10.38	20.68	7.01	81.13	0.17
8	22.00	16.55	22.73	6.47	65.70	0.36
9	30.00	22.44	32.74	12.83	57.82	0.58
15	42.38	33.31	53.57	17.51	79.79	0.36
16	47.57	47.02	77.51	22.69	107.32	0.23
2.1	76.86	36.19	130.33	50.46	85.84	0.38
2.2	59.00	50.10	105.98	37.58	103.82	0.45

### 3.2 Park Grass

**Fig. 9:**
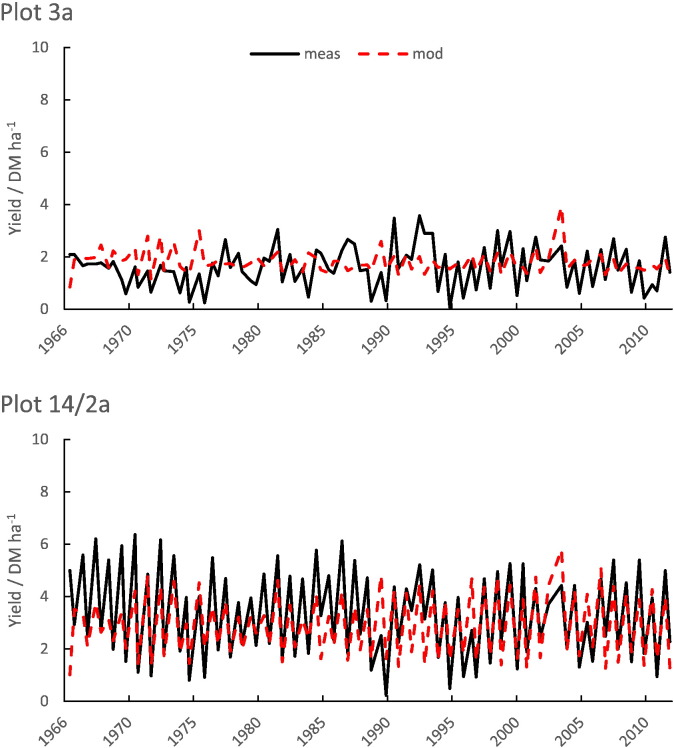
Simulated (red dashes) and measured (black lines) yields for plots 3a and 14/2a Park Grass permanent grassland experiment, showing both cuts each year.

The model captures the differences between the plots and between the first and second cuts well (Fig. 9 and Table 7). The first cut, usually taken in June, is normally higher than the second cut which is usually taken in November.

**Table 7:** Summary statistics for measured and simulated yield 1966–2012, Park Grass experiment (47 years, *n* = 93).

Plot no.	Measured		Simulated			
	Mean t ha^− 1^	Standard deviation/ t ha^− 1^	Mean t ha^− 1^	Standard deviation/ t ha^− 1^	RMSE (%)	Correlation
3a	1.61	0.78	1.79	0.43	49.91	0.28
14/2a	3.32	1.67	2.91	1.24	34.35	0.77

### 3.3 North Wyke Farm Platform.

**Fig. 10:**
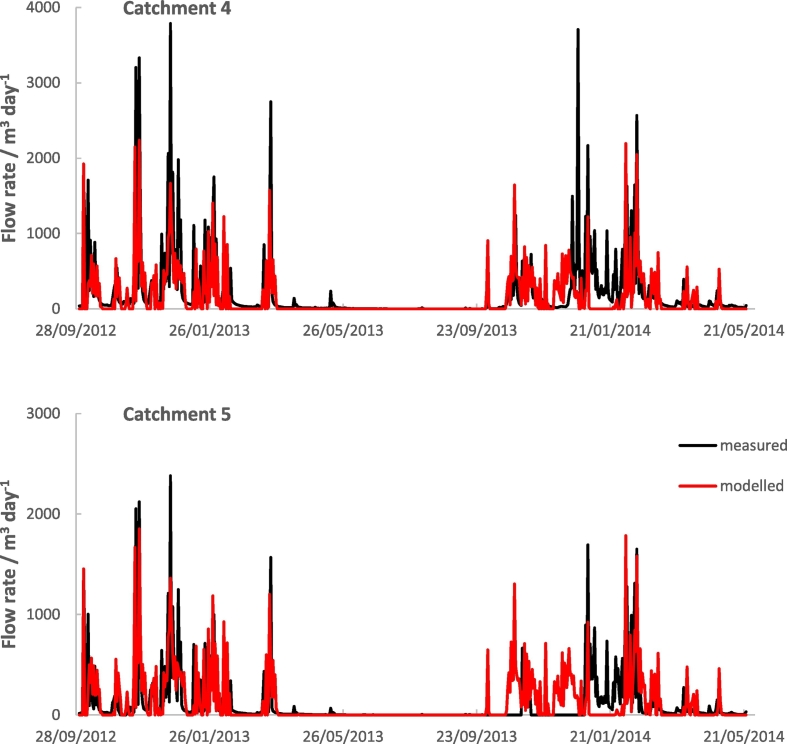
Simulated (red) and measured (black) flow rates (m^3^ day^− 1^) for catchments 4 and 5 of the North Wyke Farm Platform.

The simulation of water flow rates (m^3^ day^− 1^) for catchments 4 and 5 reflect those measured (Fig. 10 and Table 8). This is quantified by the correlations between modelled and measured (Pearson correlation, *r* = 0.57 and *r* = 0.55 respectively). The modelled water flow rate and variation are slightly smaller than the measured in each case.

**Table 8:** Summary statistics for measured and simulated flow and nitrate (kg N per catchment per day) in the drains, North Wyke Farm Platform Catchments 4 and 5.

Catchment	Flow (m^3^ day^− 1^)						Nitrate (kg N catchment^− 1^ day^− 1^)					
	Measured		Simulated				Measured		Simulated			
	Mean	Std dev	Mean	Std dev	RMSE (%)	Correlation	Mean	Std dev	Mean	Std dev	RMSE (%)	Correlation
4	213.60	457.01	147.83	315.90	180.36	0.57	0.13	0.27	0.47	1.64	1287.69	0.21
5	114.10	281.82	122.88	258.19	226.02	0.55	0.13	0.29	0.01	0.08	248.45	− 0.01

**Fig. 11:**
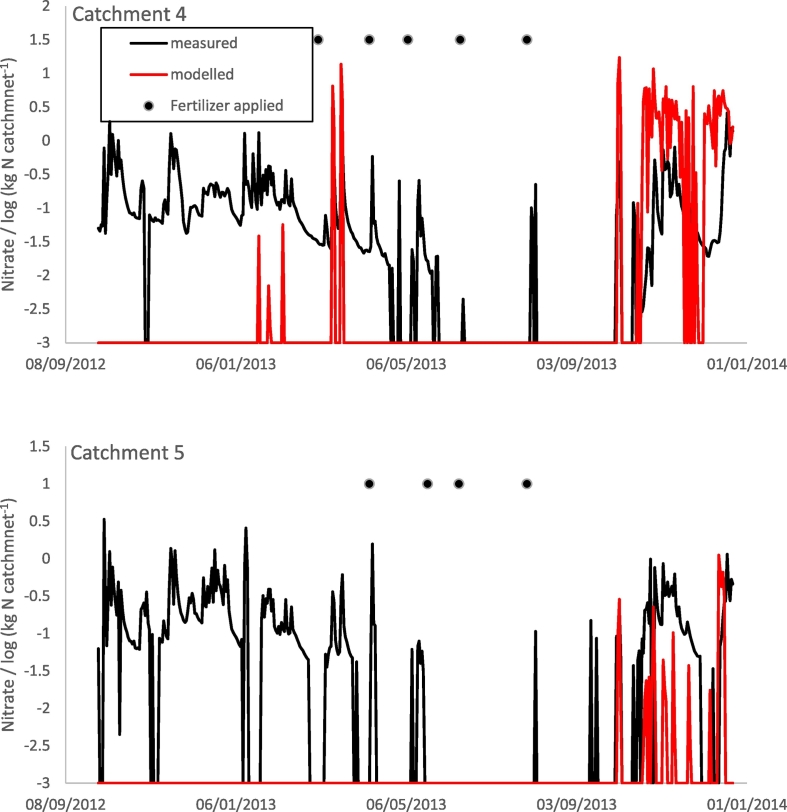
Simulated (red line) and measured (black line) log nitrate (kg N/catchment) for catchments 4 and 5 of the North Wyke Farm Platform. The black discs show when nitrogen fertilizer was applied. For details see http://www.rothamsted.ac.uk/farmplatform.

The simulation of nitrate in the drainage water over estimates nitrate for catchment 4 and under estimates it for catchment 5, but the peaks of nitrate after May 2013 broadly correspond to that which was measured (Fig. 11 and Table 8).

### 3.4 Trade offs

**Fig. 12:**
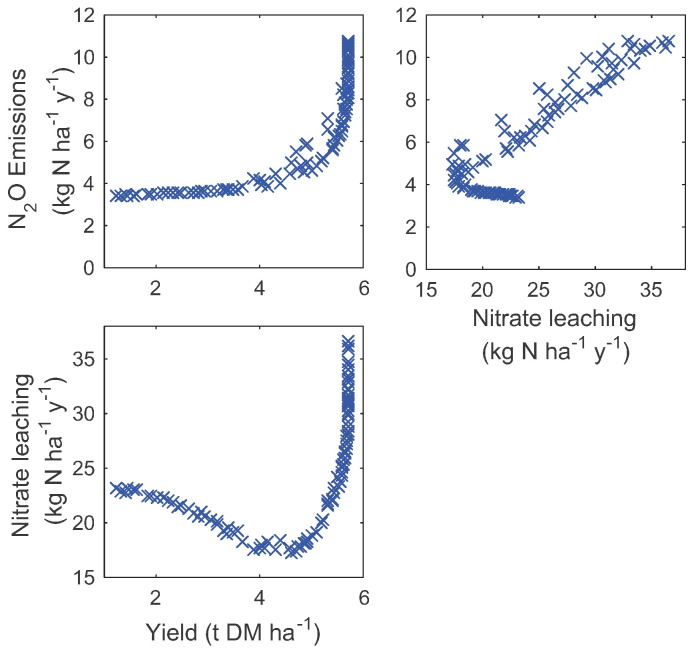
Illustrative example of use of the model to identify trade-offs between multiple objectives such as maximising yield, minimising nitrate leaching and minimising N_2_O emissions. As maximising or minimising any one of these objectives affects the others, the optimisation identifies points on a multi-dimensional frontier with Pareto optimality. On this frontier no objective can be improved upon without a detrimental effect on at least one of the other objectives. This frontier therefore represents the best trade-offs that can be achieved.

By allowing an optimisation algorithm to vary the timing and amount of a single fertilizer application, we identified the trade-offs between yield, nitrate leaching and N_2_O emissions for an illustrative example (Fig. 12). The results show that as the yield increases (due to changes in fertilizer application) the lowest possible N_2_O emissions that could be achieved simultaneously increases non-linearly. The range of fertilizer N applied to achieve these Pareto optimal objectives was 0–210 kg N ha^− 1^ y^− 1^. The N_2_O emissions reduce as a result of applying less fertilizer later in the growing season. As yield approaches its maximum, both the N_2_O emissions and the nitrate leaching increase substantially with increasing amounts of fertilizer for an increasingly marginal improvement in yield. Nitrate leaching and N_2_O emissions are synergistic throughout most of the range, however a trade-off appears as the emissions reach their minimum value, as this also results in an increase in leaching. This illustrates how an optimisation approach (e.g. minimising N_2_O) could have unintended consequences for another process (nitrate leaching), if both objectives are not considered simultaneously. The optimisation algorithm does not identify a single fertilization strategy, but highlights nonlinearities thus identifying where a small reduction in one objective could have a large benefit to another. Here, for example, the simulation indicates that the fertilizer application conditions which correspond to a moderate yield, reduce the nitrate that is available to leach from the soil substantially compared to those required for the most yield.

## 4 Discussion

We have built and used a model framework to simulate spatial and temporal interactions in agricultural landscapes. The framework allows us to explore trade-offs between production and environmental outcomes to determine strategies that could contribute to sustainable food production. It is important that the models reflect the important mechanisms that relate to production and the environment. It is also essential that the models are parsimonious and run quickly so that a large range of scenarios can be tested, perhaps in conjunction with an optimisation algorithm. Our simulations are within 25% of all the observations across multiple years and plots and this is good evidence that the model is robust and that we can use it with confidence to explore trade-offs relevant to farm and environmental management.

Simulation of wheat yields from the Broadbalk experiment and grass yields from the Park Grass experiment reproduced both the differences between plots caused by the various fertilizer rates (*ρ* > 0.78) and the observed year-to-year variation (RMSE ranging between 20.3 and 28.6% for the mineral N and FYM plots on Broadbalk and 34.3% for Park Grass, correlations were up to 0.77). According to the RMSEs, the model performed less well for the plots that received no fertilizer (plots 3 and 5 on Broadbalk and plot 3a on Park Grass) where the RMSEs were 42.6, 33.4% and 49.9% respectively. The larger values for the RMSE on the lower-yield plots to some extent result from the form of this statistic which is scaled by the reciprocal of the mean observation (i.e. the sum of the squared difference for the lower-yielding plots are scaled by larger values than the higher-yielding plots). Over the 46 years that we simulated Broadbalk, the model tended to under predict yield between 1994 and 1996 for plots with higher rates of N fertilizer applied (plots 8, 9, 15, 16, 21, 22) (Fig. 3). This is likely to be a result of excessive water stress when there was no N limitation. It was drier than normal in the three months before harvest in 1994, 1995 and 1996, this led to higher water stress during those months, and so a reduction in dry matter production.

The predictions of the variation in grain N for the Broadbalk plots were also good, with the RMSE ranging from 21.3 to 42.5% (Fig. 4, Table 3), and again illustrated the differences between plots receiving different rates of fertilizer N. For P uptake by the crop, the model performed well for most plots with RMSE between 20.2 and 29.0% for all plots except 3 and 5 which had RMSE of 89.5% and 48.3% respectively (Fig. 5 and Table 4). In the experiment applications of P stopped in 2001 due to large amounts of plant-available P in the soil, and the P measured in the grain declines noticeably in plots with larger applications of fertilizer but this is not exhibited in the model. However, this does not affect the measured grain yields (Fig. 3). The variations in simulated yield, grain N and P are approximately 50% smaller than the observed for plots 3 and 5 (for other plots the variation is proportionally more similar). This suggests that the nitrogen stress function maybe over-damping the simulated response to variation in the weather.

The modelled TOC for the Broadbalk plots fits the measured data well with the RMSE ranging from 3.1 to 13.8% (Fig. 6 and Table 5).

The model simulations of N leached from the Broadbalk plots were compared with estimates of leaching (Goulding et al., 2000), based on nitrate concentrations in drainage and soil water and calculations of drain flow. The measured concentrations of nitrate in soil water were subject to the usual large spatial variation with typical CVs of 50–90%. The simulations reflected the differences in leaching between the different amounts of N, although they tended to overestimate N leached at the largest N rates and in the driest years (Fig. 8 and Table 6). IPCC guidelines (IPCC, 1997, Del Grosso et al., 2005) assume that 30% of applied N is leached or runs off into groundwater or surface waters and this accords with our simulations of Broadbalk where approximately 31.7% of N applied is lost through leaching.

The simulation of water flow from the two North Wyke Catchments matches the pattern in the variation of water flow but the average water flow over the simulated periods was larger than that simulated, as was the variation. This suggests that our model system is buffering the water through-put in the catchment and that too much is being taken by the crop or evaporating from the system. The simulations of nitrate in drainage water on the North Wyke plots appeared to be poorer than the simulations of N losses for Broadbalk. Although the timing of peaks in nitrate towards the end of the simulation were determined well, little nitrate was simulated in the first part of the simulated time period. This was because there was very little nitrate left in the model soil profiles at the beginning of the simulated run, and during the summer period (May 2013 – September 2013) there was very little simulated discharge (see Fig. 10). An addition of nitrate on 5th March 2013 to catchment 4 increased the nitrate levels in the soil and a peak in nitrate followed. Further additions of nitrate fertilizer kept the soil nitrate in this simulation at a larger concentration than that in the catchment 5 simulation, which despite having similar levels of nitrate applied, retained less nitrate in the soil. The difference in the simulated soil nitrate between the two catchments manifests as differences in the nitrate in the drainage water in the autumn and winter of 2013 where the nitrate leached was greater for catchment 4 than for catchment 5. The simulated nitrate in the drainage water is larger than that measured for catchment 4 yet smaller for catchment 5. This suggests problems with the modelled uptake of nitrate by the grass and retention in the soil in this case, but we have no explanation for the counter-intuitive discrepancy between the measurements on the two plots. Quantifying the fate of nitrate can be difficult (Senapati et al., 2016). Recently calculated field level budgets of N from the North Wyke Farm Platform show unaccounted for losses of between 30 and 60 kg N ha^− 1^ (Misselbrook pers. comm.). This highlights the need for more research on the processes that control N transformations from micro-scale to field scale, and larger-scales. Facilities such as the North Wyke Farm Platform are ideally placed to support this kind of research. Models such as the one described here can help to identify the parts of the processes where understanding is incomplete and so can help to inform the design of experiments as well as benefit from any new understanding obtained.

Others have explored trade-offs using empirical data. For example Phalan et al. (2011) compared the effects of land sparing and land sharing on crop yields and the densities of tree and bird species across the UK, while Lamb et al. (2016) explored the need to cut greenhouse gas emissions, while increasing agricultural yields to meet the rapidly rising food demand through land sparing. Eory et al. (2013) examined the trade-offs and synergies between greenhouse gas mitigation measures and other environmental pollutants. The limitation of such empirical studies is that there is a lack of data and so it is often not possible to consider more than two factors at a time. Whilst models should always be used with caution, they do allow us to consider multiple interactions under a large range of management strategies. Used appropriately, models such as the one we present here should allow sound conclusions to be drawn on the relative impact of management strategies and might highlight unintended consequences of certain actions. Whilst the complexity of agricultural systems across the landscape could warrant a complex model, a simpler model that runs more quickly but still captures the key processes can be coupled more easily to an optimisation algorithm. This then provides the opportunity to identify the form of the synergies and trade-offs between multiple objectives at a broad and often neglected scale. Here, for example, we observe that objectives that are largely synergistic such as nitrate leaching and N_2_O emissions still exhibit a trade-off as the N_2_O emissions approach the minimum. The non-linearity in the leaching and emissions as yield increases is also clear, indicating a strong trade-off.

In order to generate frontiers such as the ones we did here (Fig. 12) an optimisation algorithm must be chosen and a set of management options that the optimisation algorithm can manipulate identified. Within an agricultural landscape, management options are numerous. For example, even considering only fertilizer applications, the timing, amount and type of multiple applications could all be included in the set of management options to be optimised. This set of options will constrain the frontier, thus care must be taken to identify a reasonable range of options, whilst keeping the number of variables that the algorithm can manipulate to a minimum. Even so, the set of options is likely to represent a complex optimisation problem, involving multiple control variables, with the risk that the algorithm may be trapped in local minima. The optimisation algorithm must be chosen and implemented to minimise this risk. In this case we chose to use non-dominated sorting combined with differential evolution. Whilst the non-dominated sorting allowed us to consider multiple-objectives, which is critical to our aim of generating trade-off curves, the differential evolution combines a genetic algorithm and a gradient based search to allow a complex control space to be explored efficiently.

Our framework includes models of crop growth, the dynamics of soil conditions and water and nutrient flows in order to quantify the trade-offs between agricultural production and environmental factors. It could be expanded to include volatilisation and biological N fixation (which should improve the simulation for certain grass and crop types). Our framework is distinct from alternative models of the agricultural landscape because it simulates multiple functions simultaneously and distinct from other models of ecosystem services (e.g. Sharps et al., 2017) because it focuses on scaling up the effect of field and farm scale management practices to landscape scale. Additional environmental factors are also relevant to the agricultural landscape and to include these the model could be expanded to include weeds, pests and diseases and aspects of biodiversity. For each new component there will be feedbacks into existing models that alter the dynamics of yield accumulation and soil nutrient status. For example, weed population dynamics will depend on the crop and the soil conditions, but in turn weeds will have a competitive effect on the crop, primarily for light, that will affect both yield and to some extent soil nutrient status (Kropff and van Laar, 1993). Our model framework is spatially explicit and simulates interactions between cells, in particular it describes the lateral flows of nutrients and water from cell to cell based on relative elevation and slope of model cell. The movement of insect pests, for example, is somewhat different as choice of destination are influenced by host plant distribution and the dispersal characteristics of the species in question. It will be straightforward to include these dispersal mechanisms within the landscape framework, see Milne et al. (2015).

The following is the supplementary data related to this article.

Fig. S1Derivation from soil measurements of the conversion between the model pools available P and non-available P. Available P is equated to Olsen P (extraction with 0.5 M sodium bicarbonate) and non-available P is equated to total P minus Olsen P. Total P was determined following aqua regia digestion. Olsen P (mg kg^− 1^) is plotted against total P (mg kg^− 1^) for soil from the Broadbalk Experiment, Rothamsted in year 2000 (P. Poulton, pers. comm.). Two regressions are fitted: to values for which total P is a) smaller  and b) greater  than the value of total P at the junction of the two regression lines. This critical value of Total P was obtained iteratively by allocating observations to each regression until the regression lines met at the assumed critical value of Total P. The values of the regression coefficients for this to occur are Slope *α*_*a*_: 0.02010; intercept *β*_*a*_: − 5.097; R^2^: 0.191; Slope *α*_*β*_: 0.1132; intercept *β*_*β*_ : − 49.27; R^2^: 0.905.Fig. S1
