# Supplementary material for: The landscape model: A model for exploring trade-offs between agricultural production and the environment
Source: Sci Total Environ. 2017 Dec 31;609:1483–99. doi: 10.1016/j.scitotenv.2017.07.193 (PMC5622278; doi:10.1016/j.scitotenv.2017.07.193)

Fig S1: Derivation from soil measurements of the conversion between the model pools available P and non-available P. Available P is equated to Olsen P (extraction with 0.5 M sodium bicarbonate) and non-available P is equated to total P minus Olsen P. Total P was determined following aqua regia digestion.

Olsen P (mg kg^-1^) is plotted against total P (mg kg^-1^) for soil from the Broadbalk Experiment, Rothamsted in year 2000 (P. Poulton, pers. comm.). Two regressions are fitted: to values for which total P is a) smaller ◆ and b) greater ◼ than the value of total P at the junction of the two regression lines. This critical value of Total P was obtained iteratively by allocating observations to each regression until the regression lines met at the assumed critical value of Total P. The values of the regression coefficients for this to occur are Slope $\alpha_{a}$: 0.02010; intercept $\beta_{a}$: $-5.097$; R^2^: 0.191; Slope $\alpha_{\beta}$: 0.1132; intercept $\beta_{\beta}:-49.27$; R^2^: 0.905.


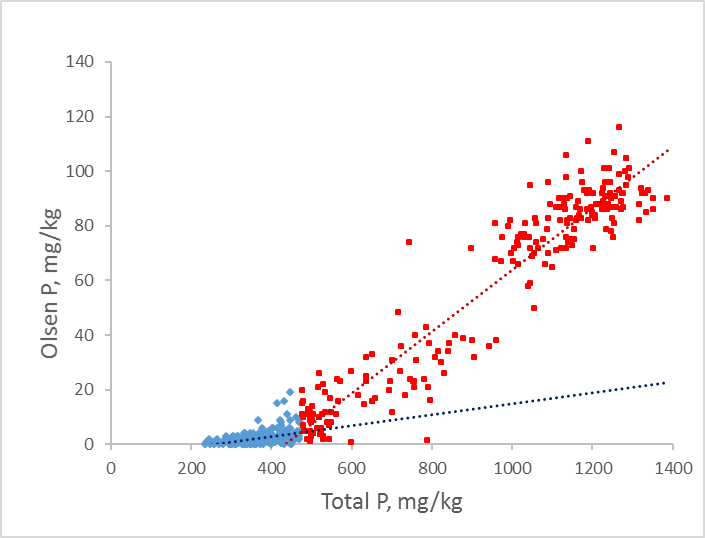

Supplement: Fig. S1 — Derivation from soil measurements of the conversion between the model pools available P and non-available P. Available P is equated to Olsen P (extraction with 0.5 M sodium bicarbonate) and non-available P is equated to total P minus Olsen P. Total P was determined following aqua regia digestion. Olsen P (mg kg− 1) is plotted against total P (mg kg− 1) for soil from the Broadbalk Experiment, Rothamsted in year 2000 (P. Poulton, pers. comm.). Two regressions are fitted: to values for which total P is a) smaller Image 2 and b) greater Image 3 than the value of total P at the junction of the two regression lines. This critical value of Total P was obtained iteratively by allocating observations to each regression until the regression lines met at the assumed critical value of Total P. The values of the regression coefficients for this to occur are Slope αa: 0.02010; intercept βa: − 5.097; R2: 0.191; Slope αβ: 0.1132; intercept ββ : − 49.27; R2: 0.905. [file mmc1.docx]
